# Non-universality of the dynamic exponent in two-dimensional random media

**DOI:** 10.1038/s41598-018-36236-z

**Published:** 2019-01-22

**Authors:** Hyun Woo Cho, Arun Yethiraj, Bong June Sung

**Affiliations:** 1 Department of Chemistry and Research Institute for Basic Science, Sogang University, Seoul, 04107 Republic of Korea; 2 Theoretical Chemistry Institute and Department of Chemistry, University of Wisconsin, Madison, Wisconsin 53706 USA

## Abstract

The diffusion of solutes in two-dimensional random media is important in diverse physical situations including the dynamics of proteins in crowded cell membranes and the adsorption on nano-structured substrates. It has generally been thought that the diffusion constant, *D*, should display universal behavior near the percolation threshold, i.e., *D* ~ (*ϕ* − *ϕ*_*c*_)^*μ*^, where *ϕ* is the area fraction of the matrix, *ϕ*_*c*_ is the value of *ϕ* at the percolation threshold, and *μ* is the dynamic exponent. The universality of *μ* is important because it implies that very different processes, such as protein diffusion in membranes and the electrical conductivity in two-dimensional networks, obey similar underlying physical principles. In this work we demonstrate, using computer simulations on a model system, that the exponent *μ* is not universal, but depends on the microscopic nature of the dynamics. We consider a hard disc that moves via random walk in a matrix of fixed hard discs and show that *μ* depends on the maximum possible displacement Δ of the mobile hard disc, ranging from 1.31 at Δ ≤ 0.1 to 2.06 for relatively large values of Δ. We also show that this behavior arises from a power-law singularity in the distribution of transition rates due to a failure of the local equilibrium approximation. The non-universal value of *μ* obeys the prediction of the renormalization group theory. Our simulations do not, however, exclude the possibility that the non-universal values of *μ* might be a crossover between two different limiting values at very large and small values of Δ. The results allow one to rationalize experiments on diffusion in two-dimensional systems.

## Introduction

The transport of a solute in heterogeneous and disordered media is relevant to a variety of systems including the protein diffusion in cells^1–8^, the electrical conductivity of polymer nanocomposites^9–14^, two dimensional metal insulator transition^15–19^, fluid flow through fractures^20–23^ and porous separation membranes^24–29^. In all these systems, the diffusion coefficient (*D*) of the solute (proteins, electrons, and analyte) scales as $D\sim {({\varphi }_{c}-\varphi )}^{\mu }$ where *ϕ* and *ϕ*_*c*_ are the area fraction of the matrix particles and its value at a critical pore percolation threshold, respectively, and *μ* is a dynamic scaling exponent^12,30–32^. It is generally thought that *μ* is a universal exponent in two dimensions (2D) with *μ* = 1.31^33–36^. In this work, we show that *μ* is not universal and establish the reasons for the non-universality in this dynamic exponent.

The universality of *μ* can be predicted by renormalization group theory. Any random matrix can be represented in terms of pores that are connected by channels and diffusion of solutes can be regarded as sequential transitions of solutes between neighboring pores. The dynamic exponent *μ*, therefore, is related to the transition rate (*W*) between neighboring pores and the distribution function *ρ*(*W*)^37–40^ as well as the structure of pore clusters. Straley established the contribution of *ρ*(*W*) to the value of *μ* using renormalization group theory^37^. If *ρ*(*W*) ~ *W*^−*α*^ for *W* → 0, $\mu =\,{\rm{\max }}\,[{\mu }^{latt.},(d-2)\nu +1/(1-\alpha )]$, where *μ*^*latt*.^ (=1.31 for 2D) is the value of *μ* for a regular lattice, *d* is the dimensionality of space, and *ν* is a universal exponent for the correlation length of the pore cluster that depends only on *d*^30–32^. In 2D, for examples, $\mu =\,{\rm{\max }}\,[1.31,1/(1-\alpha )]$, which implies that a strong power-law singularity in *ρ*(*W*) can result in a non-universal value of *μ* > 1.31.

Machta and Moore suggested that *α* should be 0 in 2D disordered media, thus concluding that *μ* should be universal with *μ* = 1.31^33^. They estimated *W* between neighbor pores by employing transition state theory (TST)^33^, which has been used successfully to calculate the rates of various reactions^41^. According to TST, the reaction rate is proportional to the ratio of the partition function of transition state and the partition function of reactant^42^. Employing this idea, they determined *W* for 2D porous media as the ratio of the channel gap size and the area of the pore^33,41,43^, and found that there should be no singularity in *ρ*(*W*) for random porous media, suggesting that $\alpha \approx 0$ and *μ* would be universal^26,33^. Simulation studies of a 2D Lorentz gas, i.e., a point particle in a matrix of hard discs, showed that *μ* = 1.31^36^, in agreement with the theoretical predictions, which supported the universal behavior of *μ* in 2D.

Recent experiments and simulation studies on various 2D systems^4,18–20,44–47^, however, reported non-universal values of *μ*, typically larger than *μ* = 1.31 ranging from 1.3 to 4.34. For example, the electron diffusion (the electric conductivity, *S*) on the surface of *n* type GA/AS systems followed the scaling relation with respect to the carrier densities (*n*) and its critical value (*n*_*c*_), i.e., *S* ~ (*n* − *n*_*c*_)^*μ*^, but the value of *μ* increased from ~1.4 to ~2.6 as temperature increased from 47 mK to 80 mK^18^. This may indicate that *μ* could be non-universal due to a strong singularity in *ρ*(*W*) even in 2D. From previous numerical results, one may find that the microscopic dynamics of tracers may play an important role in non-universal behavior of *μ* in 2D. For example, *μ* of the Brownian particles has a universal value (*μ* = 1.310)^36^, whereas *μ* = 1.82 if a tracer particle moves in a circular orbit^48^ and *μ* = 1.74 for ballistic particles in porous media^4^.

Particularly for 3D, Spanner *et al.* showed that microscopic details of the tracer dynamics can split the universality class of the dynamic exponent^8^. They considered the diffusion of the tracers in the sea of quenched hard spheres and found that ballistic and Brownian tracers had different values of *μ*. They suggested that *ρ*(*W*) should exhibit different singularity behaviors depending on the microscopic dynamics, which would result in the splitting of the universality class of *μ*. However, scientific questions remain unanswered: would such a strong singularity in *ρ*(*W*) appear even in 2D due to the variation of the microscopic dynamic details of tracers and if so, what would be a determinant for non-universal diffusion.

In this work, employing computer simulation on a model system, we find that upon a breakdown of *local equilibrium approximation*, the value of *μ* becomes non-universal even in 2D, which is attributed to a strong power-low singularity in *ρ*(*W*). Note that local equilibrium approximation is the most basic assumption of TST stating that reactants would reach a local equilibrium before it would undergo a reaction to the product^41,42^. In case of a transport in disordered media, the local equilibrium approximation implies that a solute (proteins, electrons, and analyte) in a pore (reactant) would spend a sufficient amount of time to sample thoroughly the phase space of the pore before moving to the neighbor pore (product). Therefore, in order for TST to hold, the translational relaxation time (*τ*_*T*_) of the solute should be significantly smaller than the mean-first passage time (*τ*_*MFPT*_, the inverse of *W*). In this work, we find that depending on the microscopic dynamic nature, *τ*_*T*_ could be sufficiently larger than *τ*_*MFPT*_, in which the local equilibrium approximation may not hold and *ρ*(*W*) becomes singular. This can make *μ* non-universal, interestingly, which cannot be predicted by previous studies based on TST.

## Results

**Figure 1:**
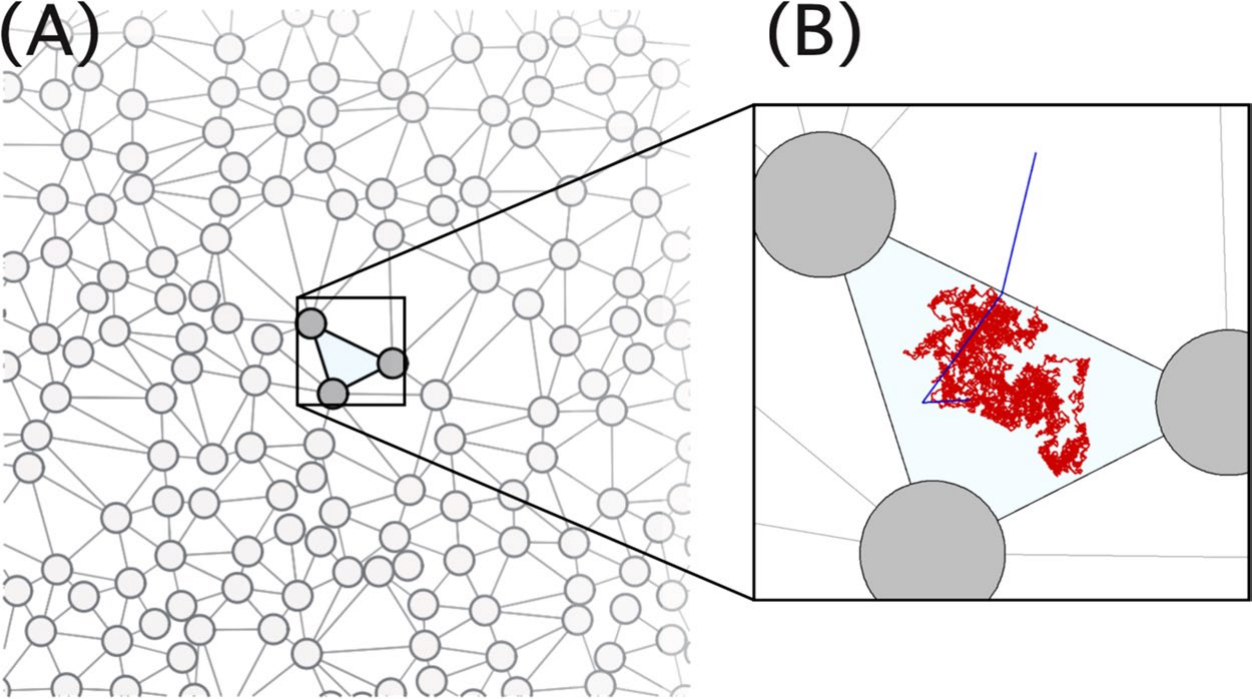
(**A**) A representative configuration of matrix particles (grey circles) of *ϕ* = 0.21. Lines are edges of Delaunay triangular cells. (**B**) A representative trajectory of a solute in a Delaunay triangle during the first passage time that it takes for the solute to escape out of the Delaunay triangle. The red and blue colors represent the trajectories for Δ = 0.01 and 2.0, respectively.

We consider the diffusion of hard discs in 2D random obstacle matrices using dynamic Mote Carlo simulations. We generate random obstacle matrices by locating and quenching hard discs at random positions without overlap in a 2D square simulation cell. Then, we locate hard discs as tracers and evolve their positions via dynamic Monte Carlo simulation (Fig. 1(A)). The tracer can move with the maximum possible displacement Δ at each trial move. We estimate the long-time diffusion coefficient (*D*) of tracers for different value of Δ and *ϕ*, where *ϕ* is the area fraction of the quenched hard discs. The values of *μ* is obtained with respect to Δ by fitting the values of *D* of given Δ to the scaling relation $D\sim {({\varphi }_{c}-\varphi )}^{\mu }$, where *ϕ*_*c*_ is *ϕ* at the percolation threshold. Detailed information on the simulation methods are described in the Methods section.

**Figure 2:**
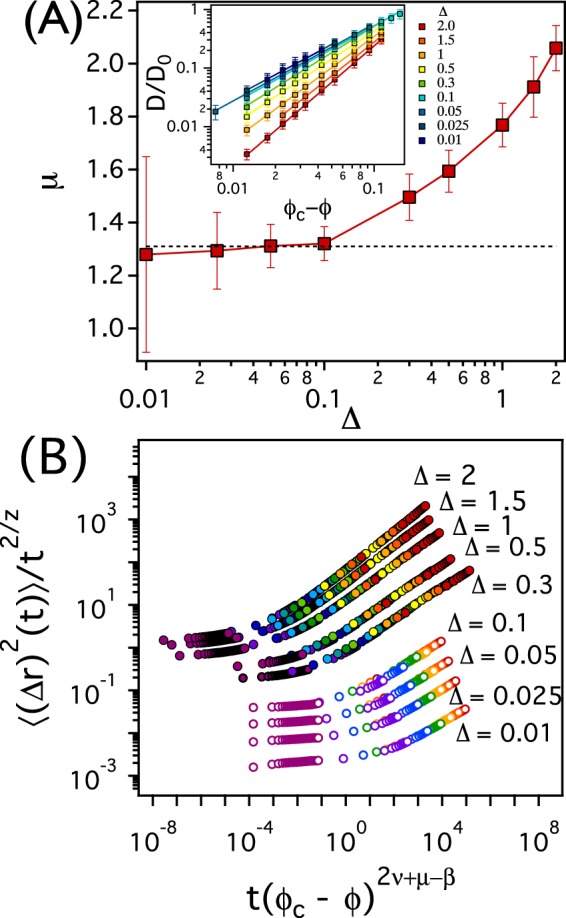
(**A**) The dynamic scaling exponent *μ* obtained from dynamic Monte Carlo simulations and 〈(Δ*r*)^2^(*t*)〉 as a function of Δ. The inset represents *D*/*D*_0_ as a function of *ϕ*_*c*_ − *ϕ* for various values of Δ. (**B**) 〈(Δ*r*)^2^(*t*)〉/*t*^2/*z*^ as a function of *t*(*ϕ*_*c*_ − *ϕ*)^2*ν*+*μ*+*β*^ for various values of Δ. The filled (open) circles represent the scaling curves for Δ from 0.3 to 2 (from 0.01 to 0.1). *μ* = 1.50, 1.59, 1.77, 1.91, and 2.06 for Δ = 0.3, 0.5, 1, 1.5, and 2, respectively. For Δ from 0.3 to 2, *ϕ* ranges from 0.1 (red markers) to 0.2075 (purple markers). And for Δ from 0.01 to 0.1, *ϕ* ranges from 0.18 (red markers) to 0.2075 (purple markers). The simulation results for 〈(Δ*r*)^2^(*t*)〉 are depicted in Figs S1 and S2 in the Supplementary Information.

The dynamic scaling exponent *μ* is a function of the parameter Δ. We estimate *μ* using *ϕ*_*c*_ = 0.2125 and the scaling relation $D\sim {({\varphi }_{c}-\varphi )}^{\mu }$. In the inset of Fig. 2(A), *D*/*D*_0_ is plotted as a function of *ϕ*_*c*_ − *ϕ* for various values of Δ, where *D*_0_ is *D* when *ϕ* = 0. When Δ ≤ 0.1, *μ* = *μ*^*latt*.^ = 1.31, which is identical to the value of *μ* in 2D regular lattice systems (Fig. 2). Interesting is, however, that as Δ increases beyond 0.1, *μ* begins to increase and deviate from *μ*^*latt*.^, which implies that *μ* is not universal in 2D but, rather, is a function of the solvent viscosity *η* through Δ.

We find that the scaling relation for the mean-square-displacement holds, if the simulation value of *μ* is used. It has been proposed that^30^,

1 $$\langle {({\rm{\Delta }}r)}^{2}(t)\rangle \sim {t}^{2/z}f(({\varphi }_{c}-\varphi ){t}^{1/(2\nu +\mu -\beta )}),$$

where, *z* = (2*ν* − *β* + *μ*)/(*ν* − *β*/2), and *β* = 5/36 in 2D. The scaling function *f*(*x*) is given by *f*(*x*) ~ *x*^*μ*^ as *x* → ∞, *f*(*x*) = *constant* at *x* = 0, and *f*(*x*) ~ (−*x*)^−2*ν*+*β*^ as *x* → −∞. If the above scaling relation is valid, 〈(Δ*r*)^2^(*t*)〉/*t*^2/*z*^ for various values of *ϕ* (≤*ϕ*_*c*_) should not only exhibit a clear plateau regime for intermediate time scales but also collapse onto a function of *t*(*ϕ*_*c*_ − *ϕ*)^2*ν*+*μ*−*β*^ at large values of *t*. As depicted in Fig. 2(B), a clear plateau regime can be seen for short time scales, and for a given value of Δ, 〈(Δ*r*)^2^(*t*)〉/*t*^2/*z*^ for different values of *ϕ* collapse onto a single curve with *μ* obtained from dynamic Monte Carlo simulations. Note in this figure that we use *μ*^*latt*.^ for Δ from 0.01 to 0.1, but non-universal values of *μ* = 1.50, 1.59, 1.77, 1.91, and 2.06 are used for Δ = 0.3, 0.5, 1, 1.5, and 2, respectively.

**Figure 3:**
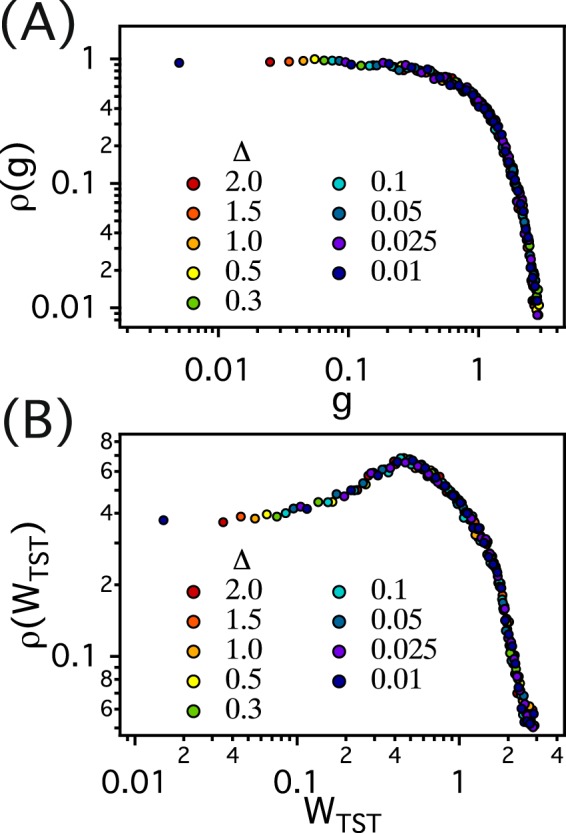
(**A**) The distribution function *ρ*(*g*) of the channel gap (g) for matrix configurations used in dynamic MC simulations for *ϕ* = 0.21 and various values of Δ. (**B**) The distribution function (*ρ*(*W*_*TST*_)) estimated by using TST and values of the channel gap (g) for various values of Δ.

The dependence of *μ* on Δ *cannot* be attributed to the gap distribution function *ρ*(*W*) because this function is, by construction, independent of Δ. We tessellate our simulation system and discretize pores in porous media into a set of Delaunay triangular cells (Fig. 1(A)) and define the channel gap size *g* as *l* − 2*σ*, where *l* is the edge of the Delaunay triangular cell and 2*σ* is the sum of the diameters of media and tracer particles. We consider up to 178348 Delaunay cells (or pores) for *ϕ* = 0.21 and estimate the distribution of *g* (*ρ*(*g*)). As shown in Fig. 3(A), *ρ*(*g*) are identical for all values of Δ.

We attribute the discrepancy between simulations and theoretical predictions to a breakdown of the transition state theory, in particular the local equilibrium approximation. According to transition state theory (TST) under the local equilibrium approximation, the reaction rate *k*_*TST*_ is proportional to the ratio of the partition function of the transition state *Q*_*TS*_ and the partition function of reactant *Q*_*reac*_, i.e. *k*_*TST*_ ~ *Q*_*TS*_/*Q*_*reac*_^41,42^. TST can be employed to estimate the transition rate (*W*) for a tracer in an initial pore to escape to its neighboring pore. In this case, the initial pore region and the channel gap between two pore regions can be regarded as the reactant and the transition state, respectively. Thus, the value (*W*_*TST*_) of *W* estimated based on TST can be expressed as *W*_*TST*_ ~ *Q*_*gap*_/*Q*_*pore*_, where *Q*_*gap*_ and *Q*_*pore*_ are the partition functions of the channel gap and the initial pore region, respectively. Since we consider the hard discs in our study, *Q*_*gap*_ and *Q*_*pore*_ should be proportional to the size of channel gap *g* and the area *A* of the initial pore region, respectively. *W*_*TST*_, then, can be determined as *W*_*TST*_ ~ *g*/*A*^33,41,43^. By employing TST, we estimate the transition rates (*W*_*TST*_) for all possible pairs of neighbor pores in our simulations. We consider up to 178348 pores for *ϕ* = 0.21 and define *W*_*TST*_ as *g*/*A*, where *A* is the area of the pore that the center of mass of the tracer particle can access. The distributions of *W*_*TST*_ (*ρ*(*W*_*TST*_)) for various Δ are depicted in Fig. 3(B). Because *ρ*(*g*) becomes constant for small values of *g* (Fig. 3(A)), *ρ*(*W*_*TST*_) also becomes non-singular and *ρ*(*W*_*TST*_) ~ *constant* with $\alpha \approx 0$ at small *W*_*TST*_ independent of Δ. According to renormalisation group theory, $\mu =\,{\rm{\max }}\,[{\mu }^{latt.},1/(1-\alpha )]$^37^. If *α* were to be 0 as suggested by TST, *μ* should be *μ*^*latt*.^ = 1.31 regardless of Δ, which is inconsistent with our dynamic MC simulation results, but is the reason why *μ* has been considered universal in 2D.

**Figure 4:**
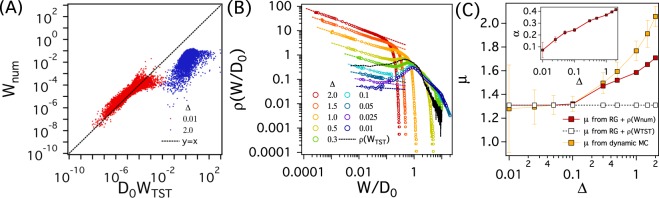
(**A**) Comparison of *W*_*num*_ with *W*_*TST*_ of individual pores for Δ = 0.01 (red points) and 0.2 (blue points). The black dashed linear line is drawn as a guide. (**B**) The distribution function (*ρ*(*W*_*num*_)) estimated numerically for various values of Δ. *ρ*(*W*_*num*_) for various Δ follows a power law relation, i.e., *ρ*(*W*) ~ *W*^−*α*^ for small *W* region (the colored dashed lines). The black dotted line represents *ρ*(*W*_*TST*_) in Fig. 3(B) Simulation results for *ρ*(*W*) for various values of Δ are depicted in Fig. S3 in the supplementary information. (**C**) Comparison of values of *μ* obtained from dynamic MC (yellow) and renormalization group theory along with *ρ*(*W*_*num*_) (red). In the inset are values of *α* obtained from *ρ*(*W*_*num*_).

A direct calculation of *ρ*(*W*) demonstrates a breakdown of the TST for large enough values of Δ. We calculate *W* numerically and its distribution (*ρ*(*W*_*num*_)) for different values of Δ. We place a tracer at a random position in each of the Delaunay cells and estimate the mean first passage time *τ*_*MFPT*_, i.e., the average time taken for the tracer to leave the cell where it was placed (Fig. 1(B)). We repeat the procedure *N*_*trial*_ (=8000) times for each pore in order to obtain the ensemble average of *τ*_*MFPT*_. The probability (*P*_*num*_(*i*,*j*), i = 1, 2, and 3, and j is the index of the pore) that the tracer escapes the *jth* pore through the *ith* side is estimated as *N*_*i*_/*N*_*trial*_. Here, *N*_*i*_ denotes the number of trajectories that the solute in the pore escapes the pore through the *ith* side of the Delaunay triangular cell. The transition rate (*W*_*num*_) of each side of the Delaunay triangular cell (or pore) is determined as ${W}_{num}=\frac{{P}_{num}}{2{\tau }_{MFPT}}$. We estimate *ρ*(*W*_*num*_) using up to 178348 pores for *ϕ* = 0.21. For a rigorous comparison between *W*_*num*_ and *W*_*TST*_, the appropriate prefactor of *W*_*TST*_ ~ *g*/*A* should be considered. For ballistic particles in random media, the prefactor (~1/*π*) can be determined by integrating the momentum space of the partition functions *Q*_*TS*_ and *Q*_*reac*_^33,41,43^. To our best knowledge, the prefactor for stochastic particles in the random media is not known exactly yet, but Kramers theory suggests that it should be comparable to *D*_0_^33,41^. Hence, in Fig. 4(A), we compare *W*_*num*_ with *D*_0_*W*_*TST*_ of each pore when Δ is 0.01 (red points) and 2.0 (blue points). The dashed line represents *y* = *x*, which is a guide line in order to compare *D*_0_*W*_*TST*_ with *W*_*num*_. In case of Δ = 0.01 where TST is supposed to work properly under the local equilibrium approximation, ${D}_{0}{W}_{TST}\approx {W}_{num}$. On the other hand, when TST fails for Δ = 2, *D*_0_*W*_*TST*_ differs significantly from *W*_*num*_.

The numerically obtained *ρ*(*W*_*num*_) is singular at small values of *W*_*num*_ unlike *ρ*(*W*_*TST*_) (Fig. 4(B)). When Δ = 0.01, *ρ*(*W*_*num*_) converges to a plateau as *W*_*num*_ is decreased, thus $\alpha \approx 0$. The overall shape of *ρ*(*W*_*TST*_) for Δ = 0.01 differs from *ρ*(*W*_*TST*_) (the black dotted line), which would be attributed to the fact that *W*_*num*_ of Δ = 0.01 deviates from *W*_*TST*_ for large values of *W* as shown in Fig. 4(A). Nevertheless, the quantitative consistency for small *W* leads to a qualitatively similar power law behaviors between *ρ*(*W*_*num*_) and *ρ*(*W*_*TST*_), i.e., *ρ*(*W*) ~ *W* ^0^. As Δ is increased, however, *ρ*(*W*_*num*_) becomes singular with *α* > 0. e estimate the values of *α* by fitting *ρ*(*W*_*num*_) at small *W*_*num*_ to a power law relation, i.e., *ρ*(*W*) ~ *W*^−*α*^ (the colored dotted lines of Fig. 4(B)), and depict *α* with respect to Δ in the inset of Fig. 4(C)). We evaluate *μ* using the renormalization group theory ($\mu =\,{\rm{\max }}\,[{\mu }^{latt.},(d-2)\nu +1/(1-\alpha )]$)^37^. Figure 4(C) depicts the values of *μ* obtained from both simulations and the renormalization group theory. *μ* estimated using the RG theory and *ρ*(*W*_*num*_) also deviates from 1.31 when Δ > 0.1. This indicates that our simulation results for *μ* and *ρ*(*W*) are consistent with RG theory.

It is, however, not conclusive from our simulation data that whether RG theory and simulation results could capture *μ* for infinitely large Δ. As shown in the inset of Fig. 4(C), *α* increases along with Δ. Therefore, one might expect from RG theory that *μ* of random media could increase indefinitely with an increase in Δ. However, as Balberg suggested, *μ* of random media might have an upper bound *μ*_*max*_ = 2*μ*^*latt*.^ as in composite materials, which was not expected by RG theory^49^. In addition, one should not exclude the possibility that our simulation results for *μ* would belong to a cross-over regime between two different universality classes. Unfortunately, the range of *μ* considered in this work is limited such that on may not confirm the behavior of *μ* for infinitely large Δ. Nevertheless, our data clearly demonstrate that *μ* can deviate from its non-universal values due to the modification of the microscopic dynamics of tracers, which cannot be captured by TST.

The failure of TST indicates that the local equilibrium approximation is not satisfied for sufficiently large values of Δ. This makes intuitive sense because for large values of Δ the solute can leave a pore without sampling the space of the pore thoroughly. In Fig. 1(B), we compare the trajectories for Δ = 0.01 (red) and 2 (blue). As shown in the figure, for small Δ, the tracer spends a sufficient amount of time in sampling the phase space of the pore, whereas for large Δ, the tracer can leave the pore only after few jumps. This snapshot clearly demonstrates that the local equilibrium approximation would hold only for small Δ.

The local equilibrium approximations may not hold for systems, that were reported to have non-universal values of *μ*. For example, the ballistic tracers in the non-overlapping random media, $\mu \simeq 1.74$^4^. The tracer mobility was so large that only after a few collisions with matrix particles the solute escaped the pore^4,43^. Likewise, the dynamics of the tracers in a circular orbit, which mimics the magneto-transport, is also non-universal, i.e., *μ* = 1.82^48^. The tracer seems to escape its neighboring pores without fully sampling the original pore in the trajectories. These indicate that the local equilibrium approximation may not hold in those systems, which is qualitatively consistent with the trajectories of large Δ in our simulation.

In our systems of random media, a channel with a larger gap is preferred to ones with small gaps more significantly than expected by TST. Note that each pore owns three channels for the tracer to take to escape the pore. TST suggests that the probability (that the solute would escape the pore through one of the three channels) should be proportional to the channel gap. For large values of Δ for which TST fails, the tracer is likely to escape only through the channel with a larger channel gap. And the tracer becomes less likely to escape through smaller channel gaps than expected by TST. This results in an increase in the number of channels with a lower value of transition rate (*W*) and singularity of *ρ*(*W*).

**Figure 5:**
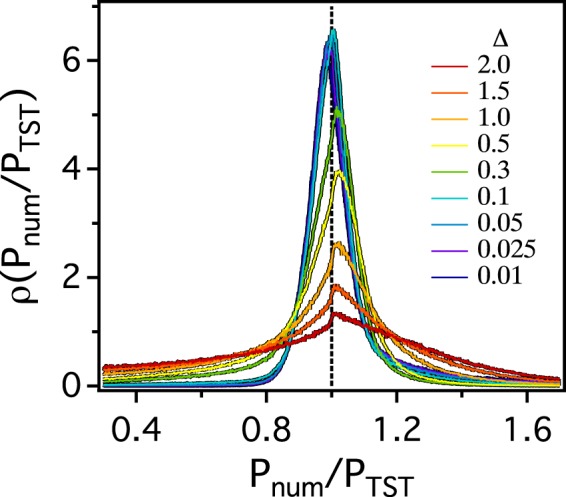
The probability distribution functions *ρ*(*P*_*num*_/*P*_*TST*_) of the ratio of *P*_*num*_/*P*_*TST*_ for various values of Δ.

In order to quantify and illustrate the failure of TST, we estimate the distribution function (*ρ*(*P*_*num*_/*P*_*TST*_)) of the ratio of the probabilities *P*_*num*_ and *P*_*TST*_. Both *P*_*num*_ and *P*_*TST*_ are probabilities that a tracer would escape a given pore through a particular channel. *P*_*TST*_ are estimated based on TST, i.e., ${P}_{TST}=\frac{{g}_{i}}{{\sum }_{i=1,3}\,{g}_{i}}$, where *g*_*i*_ is the *ith* channel gap of the pore. When gaps for all three channels of the pore are identical as in triangular lattices, for example, *P*_*TST*_ = 1/3. We estimate *P*_*num*_ numerically as discussed above for up to 178348 pores. As depicted in Fig. 5, *ρ*(*P*_*num*_/*P*_*TST*_) is sharply peaked at *P*_*num*_/*P*_*TST*_ = 1 regardless of Δ, meaning that for most pores ${P}_{TST}\approx {P}_{num}$. As Δ is increased beyond 0.1 (when *μ* begins to deviate from *μ*^*latt*.^ = 1.31), a significant amount of channels with both *P*_*num*_/*P*_*TST*_ < 0.4 and *P*_*num*_/*P*_*TST*_ > 1.6 begin to appear. This illustrates that a tracer prefers channels with larger gaps to ones with smaller gaps more significantly than expected by TST. At the same time, channels with smaller gaps are less likely to be selected by the tracer, thus increasing *ρ*(*W*_*num*_) at small values of *W*_*num*_.

Previous theoretical studies showed that when there were a significant number of channels with small gaps (*g*), *ρ*(*W*) would become singular and *μ* might deviate from *μ*^*latt*.^^37–40^. Therefore, the possible origin for the non-universal dynamic exponent would be considered a structural one. In this study, however, we show that even with identical channel gap size distribution (*ρ*(*g*)), the value of *μ* changes depending on the Δ (or the solvent viscosity). When Δ is large, the solute may not sample the phase space of pore sufficiently before the solute escape out of the pore, thus breaking the local equilibrium approximation. In such a case, TST can not hold any more and *μ* is non-universal.

## Conclusion

In conclusion, we consider the diffusion of hard discs in 2D non-overlapping random obstacle matrices using dynamic MC simulations, and show that the scaling exponent *μ* is not universal even in 2D and depends on the Δ. We obtain dynamic scaling exponent *μ* for different microscopic dynamic nature (or Δ). When Δ is small and the viscosity is large, *μ* = 1.31. As Δ increases beyond 0.1, however, *μ* begins to deviate from 1.31. We find that the non-universality of *μ* originates from the failure of local equilibrium approximation. For large values of Δ, a tracer in a pore does not sample a pore sufficiently before moving on to a neighboring pore. This results in the singularity of *ρ*(*W*) and the non-universality of *μ*. Our study indicates that even though various seemingly different systems such as cell membranes and porous separation membranes follow identical scaling relations, the dynamic exponent (*μ*) should be determined carefully because it is not, in general, universal.

## Methods

### Simulation model

**Figure 6:**
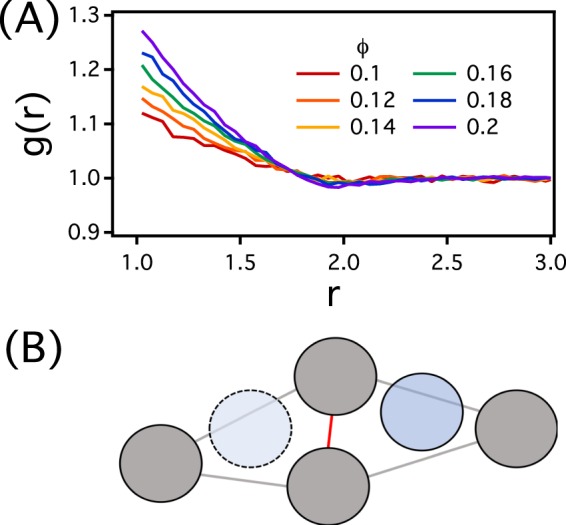
(**A**) The radial distribution function *g*(*r*) of the matrix particles for various volume fractions *ϕ*. (**B**) A schematic for a forbidden MC move in our simulation. The gray particles represent matrix particles, and bright gray and red lines represent the pore regions. The gap between two pore is drawn with red color. Note that the gap size between two pore is smaller than the diameter of the tracer particle. The blue circles of dashed and solid lines represent the tracer at current position and the tracer at a trial position, respectively.

Our system consists of hard discs (tracers) of diameter *σ* (which is the unit of length in this work) in a matrix of non-overlapping hard discs, also of diameter *σ*. The matrix particles are placed randomly and quenched in a 2D square simulation cell of linear dimension *L* with periodic boundary conditions in all directions. The dimension *L* varies from 200 to 2000 and the matrix particles are placed sequentially at random positions. If the test position overlaps with previously inserted matrix particles a new position is attempted. The area fraction ($\varphi \equiv \pi N/4{L}^{2}$) of the matrix particles ranges from 0.06 to 0.21, where *N* denotes the number of matrix particles and can be as high as one million. We depict the radial distribution function of the matrix particle for various *ϕ* in Fig. 6(A). Once the configuration of matrix particles is obtained, 10 tracers are placed at random positions such that there is no overlap between those particles. The interactions between tracers are turned off.

### Dynamic Monte Carlo Simulations

We evolve our systems using dynamic Monte Carlo (MC) simulations and a Metropolis algorithm^50^. At each attempt, we randomly choose a tracer and displace its position by *δ* for each axis. The value of *δ* is determined randomly between −Δ and Δ, where Δ represents the upper bound of *δ*. We consider various values of Δ ranging from 0.01 *σ* to 2*σ*. The move is accepted if two conditions are met: (1) there is no overlap between the tracer particle and other matrix particles, and (2) the channel gap (*g*) needs be larger than the tracer diameter (*σ*) when the tracer crosses the channel gap. The second acceptance rule is to exclude a geometrically forbidden jump of tracers between pores and ensure that the percolation threshold area fraction is independent of Δ. As shown in Fig. 6(B), according to the second acceptance rule, the tracer hard disc (the blue circle of a dashed line) cannot jump to its neighboring pore region (the blue circle of a solid line) through the gap (the distance of red line) because the gap is smaller than the tracer diameter. This forbidden jump would occur more frequently as Δ increases. Hence, we discards the trial position if the tracer jumps between the pore regions whose channel gap is smaller than the tracer size. We define the unit time *t* of our simulation as *MCS*/*N*_*tracer*_, where *MCS* and *N*_*tracer*_ are Monte Carlo steps and the number of tracers, respectively. We estimate the mean-square displacement 〈(Δ*r*)^2^(*t*)〉 and the long-time diffusion coefficient (*D*) of tracers from the Einstein relation. For each set of (*ϕ*, Δ), up to 300 matrix configurations are used to obtain the ensemble average of 〈(Δ*r*)^2^(*t*)〉 and *D*. The value of *μ* is extracted by fitting the values of *D* to the scaling relation $D\sim {({\varphi }_{c}-\varphi )}^{\mu }$.

### Determination of the percolation threshold area fraction

A precise value of *ϕ*_*c*_ is obtained as follows. For given configurations of porous media, we search for percolating pore clusters and estimate the probability *P*(*ϕ*) that a configuration of porous media would have a percolating pore cluster. *P*(*ϕ*) is determined as the ratio of the number of the configurations with percolating pore clusters to the total number of configurations. We use up to 1000 configurations for each value of *ϕ*. For a very large value of $\varphi \gg {\varphi }_{c}$, there are hardly any percolating pore clusters ($P(\varphi )\approx 0$). As *ϕ* decreases, percolating pore clusters are more likely. At sufficiently small values of *ϕ*, there are always percolating pore clusters for any configuration ($P(\varphi )\approx 1$). In the thermodynamic limit with *L* = ∞, *P* changes discontinuously from 0 to 1 at *ϕ* = *ϕ*_*c*_^30,31^. For finite systems as in our simulations, however, *P* decreases continuously with *ϕ*, and *ϕ*_*c*_ is defined as the value of *ϕ* at *P* = 0.5. By fitting *P*(*ϕ*) to a hyperbolic tangent function $P(\varphi ,L)=\frac{1}{2}[1+\,\tanh \,[({\varphi }_{c}(L)-\varphi )]/{\rm{\Delta }}\varphi ]$, we also calculate *ϕ*_*c*_(*L*)^51^. Here, *ϕ*_*c*_(*L*) and Δ*ϕ* are fitting parameters. The difference between *ϕ*_*c*_ and *ϕ*_*c*_(*L*) obeys a scaling relation, i.e., ${\varphi }_{c}-{\varphi }_{c}(L)\sim {L}^{-1/\nu }$, where *ν* is the scaling exponent for the correlation length, and is 4/3 in 2D^31^. By using the scaling relation, we find that *ϕ*_*c*_ = 0.2125 ± 0.0009.

## Electronic supplementary material

Supplementary Information

Supplementary data for MSD and the transition rate distribution
