# Supplementary material for: Non-universality of the dynamic exponent in two-dimensional random media
Source: Sci Rep. 2019 Jan 22;9:251. doi: 10.1038/s41598-018-36236-z (PMC6342955; doi:10.1038/s41598-018-36236-z)
Supplement: Supplementary file 1 — Supplementary Information [file 41598_2018_36236_MOESM1_ESM.pdf]

Supplementary Information for Non-universality of the dynamic exponent in two-dimensional random media

Hyun Woo Cho[1,2], Arun Yethiraj[2] and Bong June Sung[1*]

[1] *Department of Chemistry and Research Institute for Basic Science, Sogang University, Seoul 121-742, Republic of Korea*

[2] *Theoretical Chemistry Institute and Department of Chemistry, University of Wisconsin, Madison, Wisconsin 53706, USA*

(Dated: November 12, 2018)

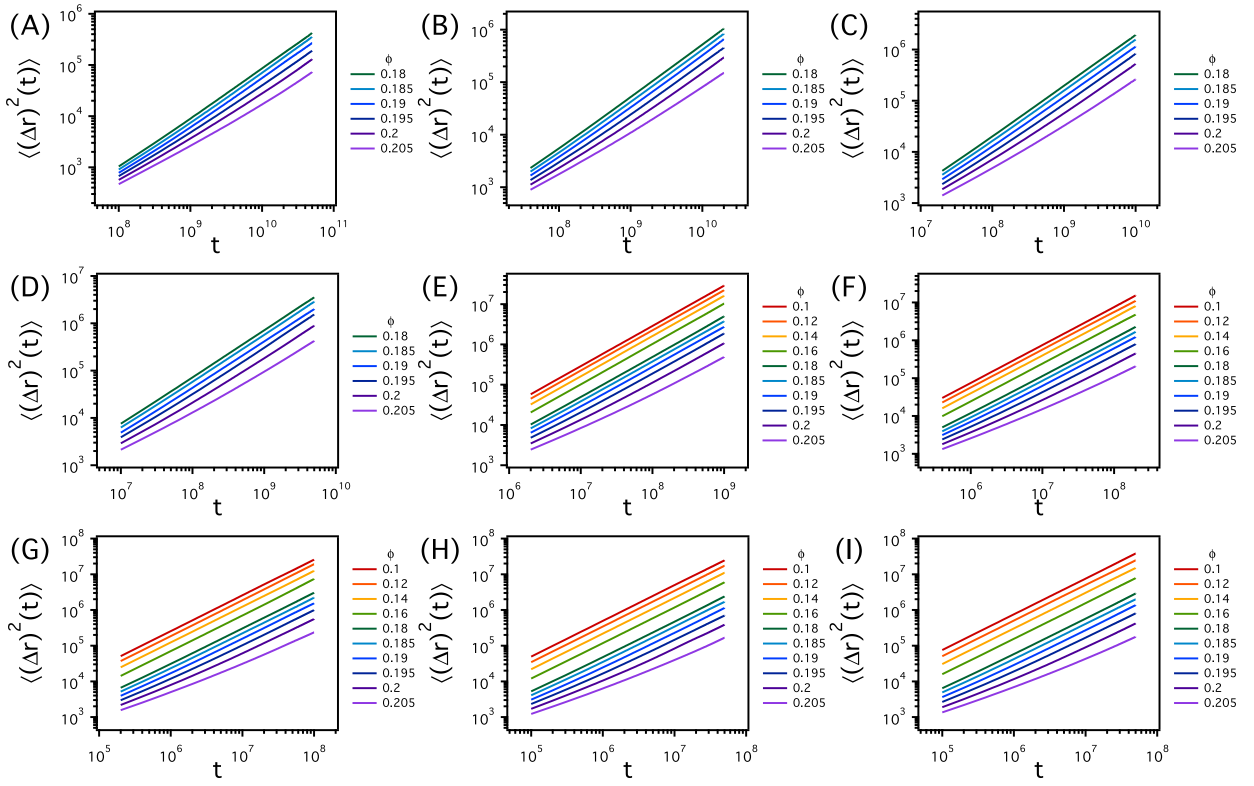

FIG. S1. Simulation results for the mean squared displacement ($\langle(\Delta r)^2(t)\rangle$) for various values of ϕ and Δ used in Figure 2(B) of the main text. The panels (A), (B), (C), (D), (E), (F), (G), (H) and (I) correspond to $\langle(\Delta r)^2(t)\rangle$'s for $\Delta = 0.01$, 0.025, 0.05, 0.1, 0.3, 0.5, 1.0, 1.5, and 2.0, respectively.

I. THE MEAN-SQUARE DISPLACEMENT ($\langle(\Delta r)^2(t)\rangle$)

The simulation results for the mean-square displacement ($\langle(\Delta r)^2(t)\rangle$) of tracers are used to determine the diffusion coefficient D and the dynamic exponent (μ), and to investigate the scaling relation ($\langle(\Delta r)^2(t)\rangle \sim t^{2/z} f((\phi_c - \phi)t^{1/(2\nu+\mu-\beta)})$) in the Figure 2 of the main manuscript. In this Supplementary Information, we present the simulation results for $\langle(\Delta r)^2(t)\rangle$ for all the sets of (Δ, ϕ) (Figure and Figure S2). Supplementary data for $\langle(\Delta r)^2(t)\rangle$'s are also provided.

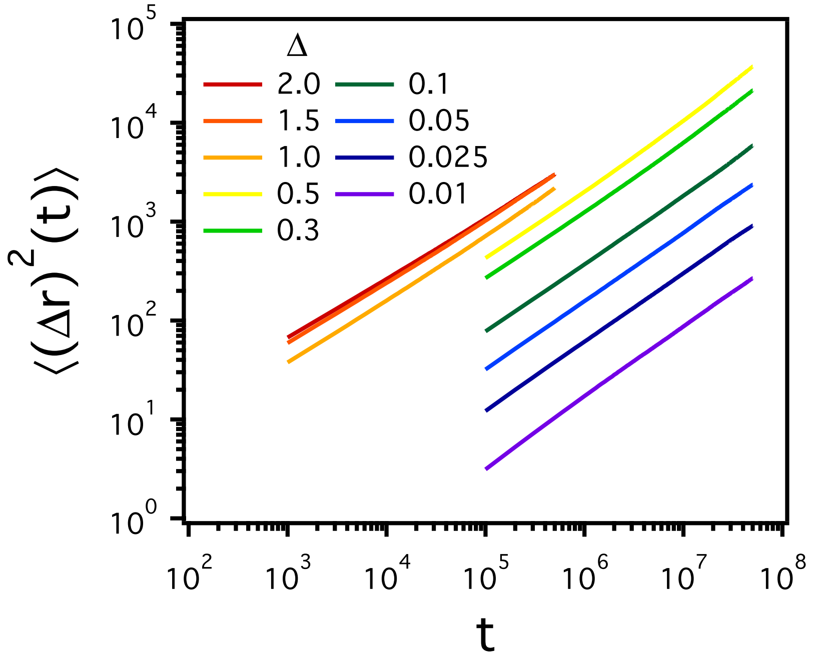

FIG. S2. $\langle(\Delta r)^2(t)\rangle$ of various values of Δ for the strong subdiffusive regime ($\phi = 0.2075$) considered in Figure 2(B) of the main text.

II. THE TRANSITION RATE DISTRIBUTION ($\rho(W)$)

In the Figure 4(B) of the main text, we compare the distribution functions ($\rho(W/D_0)$) of the rescaled transition rate (W/D_0) for different values of Δ with that of the transition state theory. In this Supplementary Information, we provide the distribution functions ($\rho(W)$) of the transition rate (W) that are used to obtain the Figure 4(B) of the main text. Supplementary data for $\rho(W)$ are also provided.

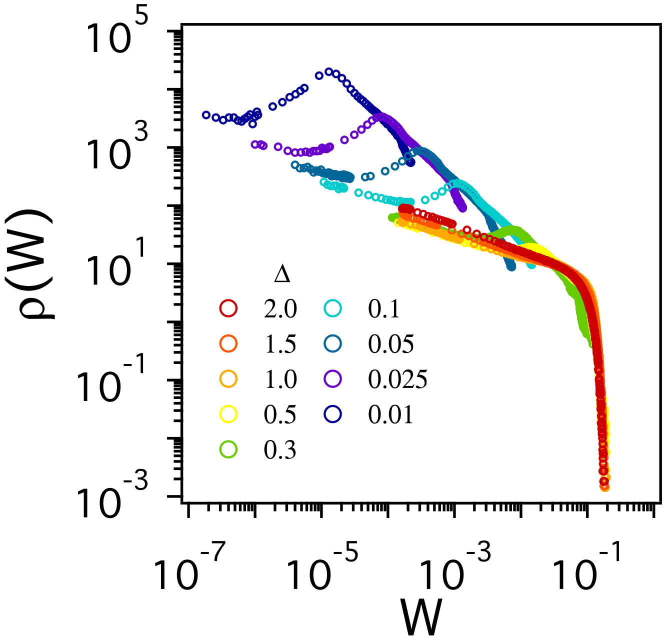

FIG. S3. The distribution function ($\rho(W)$) of the transition rate W for various values of Δ. The corresponding distribution of the reduced transition rate W/D_0 is depicted in Figure 4(B) in the main text.
